# Green synthesis of copper oxide nanoparticles and its efficiency in degradation of rifampicin antibiotic

**DOI:** 10.1038/s41598-023-41119-z

**Published:** 2023-08-28

**Authors:** Dennis Mwanza Nzilu, Edwin Shigwenya Madivoli, David Sujee Makhanu, Sammy Indire Wanakai, Gideon Kirui Kiprono, Patrick Gachoki Kareru

**Affiliations:** 1 Chemistry Department, Jomo Kenyatta University of Agriculture and Technology, P.O. Box 62000, 00200 Nairobi, Kenya; 2 Department of Biological and Physical Sciences, Karatina University, P.O. Box 1957-10101, Karatina, Kenya

**Keywords:** Environmental sciences, Chemistry, Nanoscience and technology

## Abstract

In recent ages, green nanotechnology has gained attraction in the synthesis of metallic nanoparticles due to their cost-effectiveness, simple preparation steps, and environmentally-friendly. In the present study, copper oxide nanoparticles (CuO NPs) were prepared using *Parthenium hysterophorus* whole plant aqueous extract as a reducing, stabilizing, and capping agent. The CuO NPs were characterized via UV–Vis Spectroscopy, Fourier Transform Infrared Spectroscopy (FTIR), powder X-Ray Diffraction (XRD), Scanning Electron Microscopy (SEM), Transmission Electron Microscopy (TEM), and Dynamic Light Scattering (DLS). The UV–Vis spectra of CuO NPs showed a surface plasmonic resonance band to occur at 340 nm. FTIR analysis revealed the presence of secondary metabolites on the surface of CuO NPs, with a characteristic Cu–O stretching band being identified at 522 cm^−1^. Scanning electron micrographs and transmission electron micrographs showed that CuO NPs were nearly spherical, with an average particle of 59.99 nm obtained from the SEM micrograph. The monoclinic crystalline structure of CuO NPs was confirmed using XRD, and crystallite size calculated using the Scherrer-Debye equation was found to be 31.58 nm. DLS showed the presence of nanoparticle agglomeration, which revealed uniformity of the CuO NPs. Furthermore, the degradation ability of biosynthesized nanoparticles was investigated against rifampicin antibiotic. The results showed that the optimum degradation efficiency of rifampicin at 98.43% was obtained at 65℃ temperature, 50 mg dosage of CuO NPs, 10 mg/L concentration of rifampicin solution, and rifampicin solution at pH 2 in 8 min. From this study, it can be concluded that CuO NPs synthesized from *Parthenium hysterophorus* aqueous extract are promising in the remediation of environmental pollution from antibiotics. In this light, the study reports that *Parthenium hysterophorus*-mediated green synthesis of CuO NPs can effectively address environmental pollution in cost-effective, eco-friendly, and sustainable ways.

## Introduction

Water shortage remains one of the global challenges affecting many of the world's population as it is documented that about 26% (2 billion people) lack access to safe drinking water per the UNESCO 2023 report^1^. The report further indicates that 2–3 billion people worldwide experience water shortage, with water scarcity projected to increase in the coming years. It is reported that about half of the global population is at risk of experiencing water scarcity^2^. A report published during Africa Health Agenda International Conference (AHAIC2023) revealed that climatic changes have worsened water scarcity challenges in Africa^3^. Climatic changes affect terrestrial water storage, further exacerbating water scarcity and leading to a global water crisis. Amidst the water shortage crisis, water pollution from active pharmaceutical compounds (APIs), such as antibiotics classified as emerging pollutants, continues to rise^3–5^. Pathways for antibiotics into the environment (soil or surface water) are infiltration from wastewater treatment plants and domestic discharge of human excretion^6–8^. These antibiotics exhibit detrimental effects on humans and water ecosystems, attributed to their higher concentrations above the predicted environmental concentration^8^. The existence of antibiotics in the environment propagates antimicrobial resistance^9–11^, and their low concentrations are difficult to remove using conventional wastewater treatment plants^12^. The World Health Organization declared this resistance a public health crisis, threatening modalities to treat the increasing disease burden^13^. Another potential harm exhibited by antibiotics in the environment results from their interference with physiological processes when absorbed by plants hence a propagation of ecotoxicity effect^7^.

Rifampicin is one of the antibiotics prescribed to treat tuberculosis (TB), with a global disease burden of about 10.6 million people^13–14^. In developing countries, where minimal wastewater treatment plants exist, these antibiotics, which are not fully assimilated into the body, have been observed in surface water^16^. Conventional methods to remove various wastewater antibiotics include physical, chemical, and biological methods^17^. However, these methods have limitations due to their financially intensive nature, high power consumption, and ineffectiveness in completely removing these pollutants^17,18^. To this end, researchers are considering embracing nanoparticles as catalysts for removing some of these pollutants due to their ability to find multiple applications^19–21^. These nanomaterials exhibit improved physicochemical properties than their large materials. For instance, metal nanoparticles used in the adsorption and degradation of pollutants have enhanced surface area to volume ratio and chemical stability compared to their bulk counterparts^22^. The increased surface area strengthens the removal capacity of the antibiotic into active sites of the nanoparticles^20,21, 23^. Several methods exist to synthesize metallic nanoparticles, including physical, chemical, and biological methods^19,24^. Some chemical and physical methods used to synthesize nanoparticles include sonochemistry, co-precipitation, solvothermal, pyrolysis, thermal hydrolysis, ball milling, and sol–gel^24–26^. However, chemical and physical methods for synthesizing nanoparticles have drawbacks due to their expensive nature, are time-consuming, energy inefficient, and produce toxic products making them non-environmental friendly^24,26–28^. Due to limitations associated with chemical and physical methods, researchers have recently produced eco-friendly nanoparticles by using organisms such as algae, bacteria, fungi, yeast, and plants to produce nanoparticles^20,21, 26^. The use of microorganisms such as fungi and bacteria has limitations in reducing metal ions due to their long synthesis time compared to plant-mediated synthesis^29^.

In particular, the green synthesis method utilizing plant extracts as reducing, stabilizing, and capping agents in the formation of nanoparticles is an alternative to eliminating the challenges associated with the abovementioned methods and promoting environmentally friendly chemistry^19,30, 31^. The green synthesis method is cost-effective, rapid, environmentally friendly, and sustainable since plant materials are available for large-scale nanoparticle production^26–28, 32, 33^. CuO NPs have been previously prepared using plant extracts. CuO NPs were synthesized using *Catha edulis* leaf extracts as reducing and capping agents^34^. In another study, CuO NPs were synthesized using *Eucalyptus Globulus* leaf extracts for adsorption studies^35^. *Celastrus paniculatus* was also used to prepare CuO NPs using the secondary metabolites in its leaf extracts^36^. Another study obtained CuO NPs using leaf extracts of the *Justicia schimperiana* plant^37^. Using plant extracts to synthesize CuO NPs has been proven to be cost-effective, fast, simple, and environmentally friendly^38^.

Table 1 below summarizes some of the nanoparticles or methodologies employed in the degradation of antibiotics.

**Table 1:** Overview of nanoparticles that have been employed in the degradation of antibiotics.

Removal method	Pollutant	Degradation or removal efficiency (%)	Reaction conditions	References
Superparamagnetic iron oxide nanoparticles	Tetracycline	40	UV/Vis light, 60 min	^39^
Fenton-Like reaction Fe_3_O_4_–H_2_O_2_ system	Ofloxacin	79.3	25 mL/L H_2_O_2,_ pH 4.2, 5 mg/L ofloxacin, 26 °C, 72 h	^40^
Electro-photocatalytic degradation using calcium titanate	Amoxicillin	79	100 mg/L amoxicillin solution, 0.5 g calcium titanate, pH 3 120 min, 318.5 K	^41^
Iron nanoparticles	Rifampicin	87.7	50 mg FeNPs, H_2_O_2_/pH 12	^11^
Titanium dioxide nanoparticles	Ciprofloxacin	92.81	1 g/L TiO_2_ immobilized on glass plate, pH 5, 3 mg/L ciprofloxacin, 105 min	^42^
Titanium dioxide nanoparticles	Ciprofloxacin	87.95	40 mg/L TiO_2_, 5 mg/L ciprofloxacin, pH 5, 100 min	^43^
ZnO@SiO_2_	Amoxicillin	90	10 mg/L amoxicillin, 25 mg/mL ZnO@SiO_2_, pH 8, 90 min	^44^

A common concern for nanoparticles used for wastewater treatment is their toxicity, ecotoxicity, and cytotoxicity nature^45^. The release of CuO NPs into the environment after the degradation process induces toxicity to organisms by oxidative stress responses and is affected by various factors such as shape, size, and particle concentration^45^. Therefore, in large-scale wastewater treatment, it would be challenging to identify the ultimate effect of CuO NPs after their release into the environment following a degradation process. Another consideration relates to understanding the fate of degradation products after their release into the environment. Hence, studying the ecotoxicity of CuO NPs is an ongoing area in research since there is little information available on human exposure data of nanoparticles contained in treated water^46^.

*Parthenium hysterophorus* is an invasive weed, which is much-branched and short-lived and observed to grow in many parts of the world. It belongs to the Asteraceae family. It can grow up to 1.5 m tall. It can grow in cultivated and agricultural lands and along the roadsides. It is reported to be a host of crop plant diseases and causing dermatitis in animals and humans^47^. The plant has been used as a medicinal agent and bioherbicide^48–50^. Secondary metabolites contained in *Parthenium hysterophorus* include saponins, major and trace oils, flavonoids, alkaloids, terpenoids, phenols, and tannins which can reduce ${\mathrm{Cu}}^{2+}$ to zero-valent species ${\mathrm{Cu}}^{0}$^51^. The availability of the plant makes its sustainability and scalability potential in the production of CuO NPs for large-scale wastewater treatment and removal of antibiotics feasible.

In this study, we sought to synthesize CuO NPs using *Parthenium hysterophorus* whole plant aqueous extract, and the prepared nanoparticles were characterized using UV–Vis spectroscopy, FTIR spectroscopy, XRD, TEM, SEM, and DLS techniques. The CuO NPs were then assessed on their ability to degrade rifampicin antibiotics. The degradation of rifampicin antibiotic using CuO NPs was also investigated by varying temperature, pH, the dosage of nanoparticles, concentration of the antibiotic solutions, and reaction time. To the best of our knowledge, this is the first study reporting degradation of rifampicin antibiotic using CuO NPs prepared from *Parthenium hysterophorus* whole plant aqueous extracts.

## Materials and methods

### Collection and preparation of samples

Fresh *Parthenium hysterophorus* plant was collected from Kalimoni, Juja, Kiambu County in Kenya. It was identified and authenticated by Mr. John Kamau Muchuku, a Botanist from the Department of Botany, Jomo Kenyatta University of Agriculture and Technology (JKUAT), and a voucher specimen deposited in the JKUAT Botany Herbarium with the accession number DMN-JKUATBH/001/2023A-C. The sample was thoroughly washed with running tap water, rinsed with distilled water, and shed-dried at room temperature for 2 weeks. The dry sample was then ground using a milling machine to a fine powder.

### Extraction of secondary metabolites

The procedure for aqueous extraction of *Parthenium hysterophorus* was adopted from Ref.^52^, in which 20 g of the plant sample was dissolved in 200 mL of distilled water. The mixture was heated in a hot plate at 40 °C for 45 min with constant stirring. The extract was obtained by filtration using Whatman No. 1 filter paper. The extract was then used to synthesize copper oxide nanoparticles^53^.

### Green synthesis of copper oxide nanoparticles using parthenium aqueous extract

Synthesis of copper oxide nanoparticles utilized copper (II) sulphate as a metal precursor. 0.01 M CuSO_4_ solution was prepared by dissolving 1.59 g in 100 mL of distilled water. The metal solution was then mixed with parthenium aqueous extract in a ratio of 1:4, with the formation of nanoparticles being monitored by a color change and UV–Vis spectrophotometric measurement. The colloidal solution was centrifuged at 4000 rpm for 15 min, washed several times, and the nanoparticles dried in an oven at 80 °C^54^.

**Figure 1:**
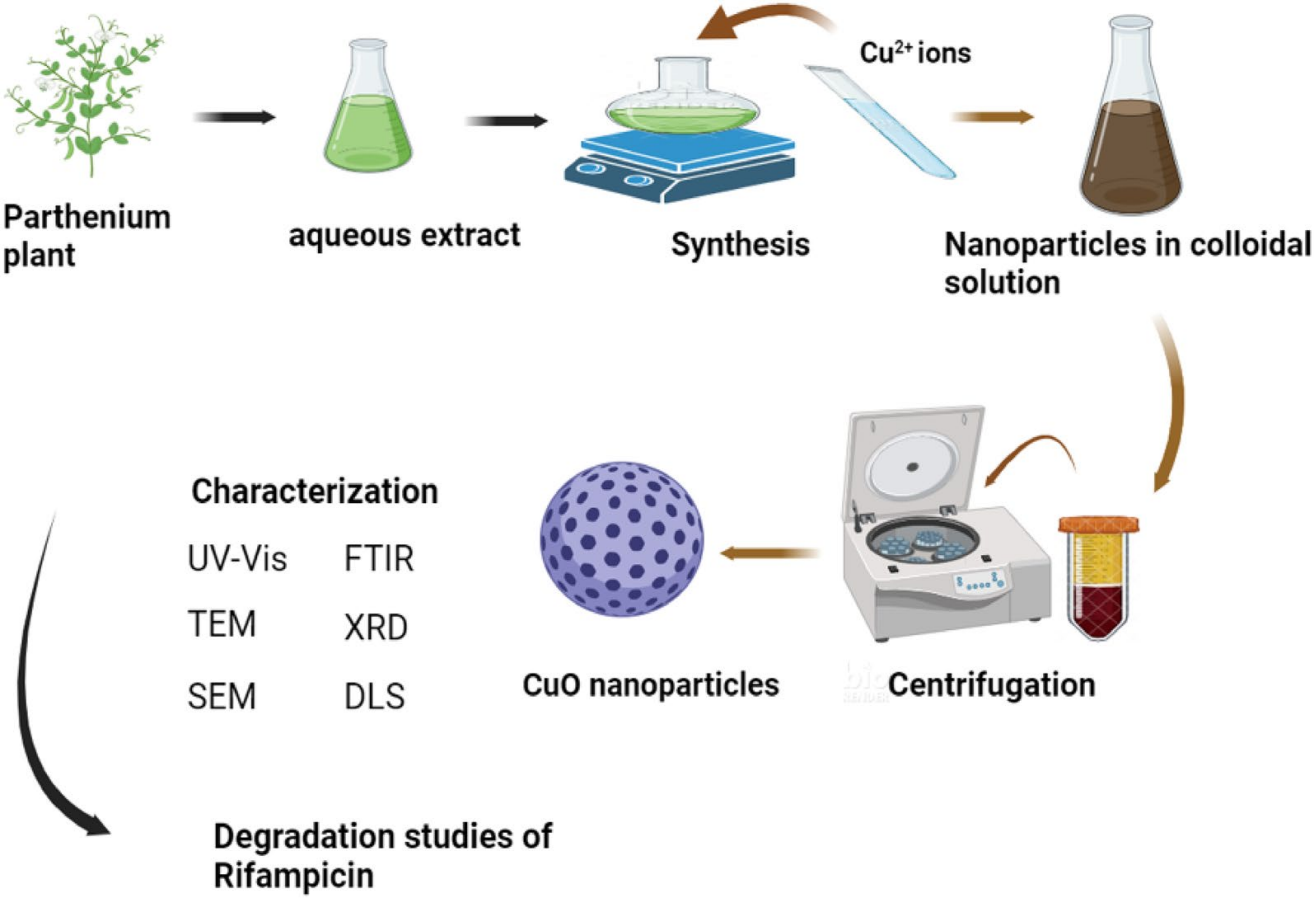
Schematic diagram showing the synthesis of CuO NPs from *Parthenium hysterophorus* aqueous extract, characterization, and use in degradation studies. Created in BioRender.com.

Figure 1 shows a schematic diagram of the synthesis of CuO NPs using *Parthenium hysterophorus* whole plant aqueous extract.

### Characterization of copper oxide nanoparticles

The synthesized nanoparticles were characterized using a Shimadzu UV-1800 UV–Vis spectrophotometer (Shimadzu, Japan) to confirm their surface plasmonic resonance at 200–800 nm wavelengths. The IR spectra of the CuO nanoparticles were acquired in the frequency range of 4000–400 cm^−1^ using a Bruker Tensor II FT-IR spectrophotometer (Bruker, Ettlingen, Germany)^55,56^. The X-ray diffractograms were obtained using a Bruker D8 Advance Diffractometer (Bruker, Ettlingen, Germany) with a copper tube operating under a voltage and current of 40 kV and 40 mA. The samples were irradiated with a monochromatic CuKα radiation of 0.1542 nm, and the diffractograms acquired between 2θ values of 5°–90° at 0.05° intervals with a measurement time of 1 s per 2θ intervals. The nanoparticles' crystalline size was calculated using Debye Scherrer's equation

$$D=\left(\frac{K\lambda }{\beta hlcos\theta }\right),$$

where D is the average particle size (nm), K is a constant equal to 0.94, λ is the wavelength of X-ray radiation, β is full-width at half maximum of the peak in radians, and theta is the diffraction angle (degree). Morphological analysis of the nanocomposite was observed using the Tescan Mira3 LM FE scanning electron microscope (Tescan, Brno—Kohoutovice, Czech Republic) operating under an accelerating voltage of 3 kV. The samples were sputter coated with 4 nm gold before analysis to avoid charging using AGB7340 Agar Sputter Coater (Agar Scientific, Essex, United Kingdom)^55,57^. TEM analysis was performed on a Tecnai G2 Spirit (ThermoFischer Scientific, Oregon USA) under an operating voltage of 120 kV equipped with veleta 2048 × 2048 wide angle and Eagle 4096 × 4096 bottom mount detectors. The dry samples were suspended in ultrapure water (Barnstead Genpure, Thermoscientific, Germany), ultra-sonicated to obtain a solution that was drop casted on 300 mesh carbon films before analysis^55,57^. To determine the particle size distribution and polydispersity index of the suspended nanoparticles, the metallic nanoparticles were resuspended in ultrapure water (18 MΩ cm Barnstead Genpure UV-TOC, Thermoscientific, Germany) and ultrasonicated to obtain a solution of suspended nanoparticles. The solutions were then filtered through 0.25 µM PTFE syringes into glass vials, and 45 µL of each solution was then transferred onto quartz cuvettes before analysis. The particle size distribution and polydispersity index were then measured using a Bechman Coulter DelsaMax pro dynamic light scattering analyzer (Beckman Coulter, Indianapolis, United States)^58,59^.

### Degradation studies

To determine the efficiency of CuO nanoparticles in the degradation of rifampicin antibiotic, degradation studies were conducted while varying pH, degradation time, the concentration of the antibiotic, temperature, and nanoparticles dose. Rifampicin solution was prepared according to Ref.^11^ in which 0.0025 g of rifampicin was dissolved in 1 mL methanol and 10 mL distilled water and the contents were transferred into a 250 mL volumetric flask^11,60^. The resultant rifampicin solution (10 mg/L) was used, and the degradation efficiency was calculated using Eq. (1)^36^

1 $$\% D\left(Degradation \right)=\frac{\left(Ao-At\right)}{Ao}\times 100,$$

where A_o,_ the initial absorbance of rifampicin, and A_t,_ the absorbance of rifampicin after time, t.

### Ethics approval

The plant materials were collected and used according to the national regulations. The utilization of this plant species for experimental purposes does not require any special permit. All methods used for this study comply with relevant institutional, national, and international guidelines and legislation. The authors recognize that the collection of plant species might be subject to guidelines established under the IUCN Policy Statement on Research Involving Species at Risk of Extinction Protocols. However, our search from the IUCN database found that *Parthenium hysterophorus* is not Red Listed or classified as a threatened species.

## Results and discussion

### UV–Vis spectral analysis

**Figure 2:**
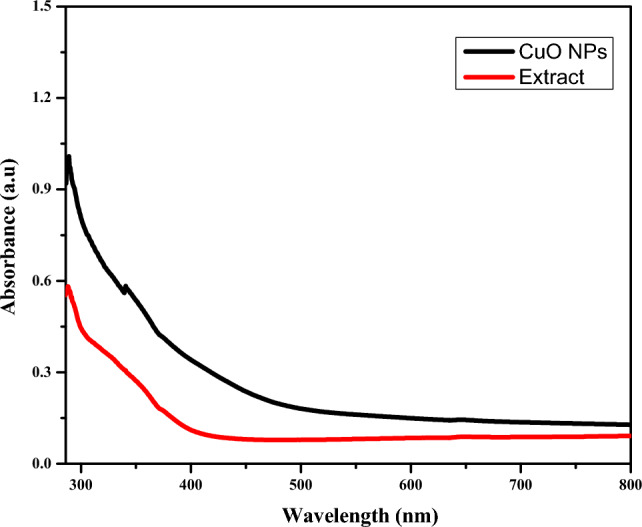
UV–Vis spectra of extract and CuO NPs.

The formation of CuO NPs was followed through measurement of its SPR peak using a UV–Vis spectrophotometer, and the results are depicted in Fig. 2.

As shown in Fig. 2, the maximum absorption peak occurred at 340 nm, attributed to the SPR absorption band of CuO NPs. The SPR absorption band of CuO NPs at 340 nm confirmed the formation of CuO NPs^61^. As the wavelength increased, the absorbance intensity decreased, indicating that formation did not occur at a large wavelength. CuO NPs formed in agreement with studies that proposed that CuO NPs formed between 200 and 350 nm^62^.

### Fourier transform infrared spectroscopy analysis

**Figure 3:**
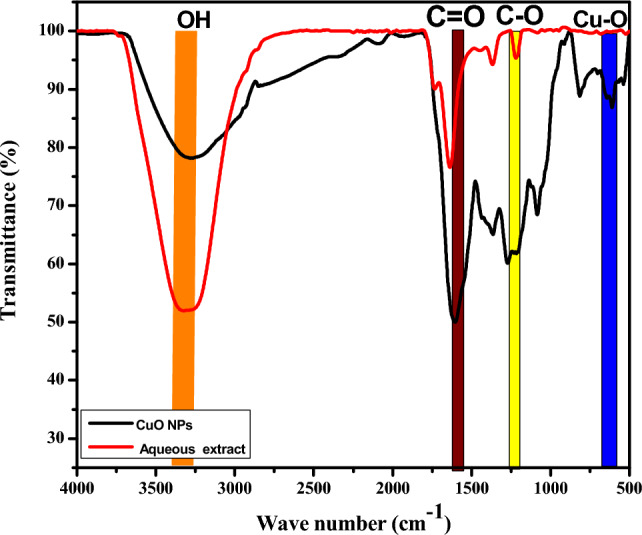
IR spectra of *Parthenium hysterophorus* aqueous extract and CuO NPs.

The functional groups present in *Parthenium hysterophorus* aqueous extract and CuO NPs were identified using FTIR spectrometry in the range of 4000–400 cm^−1^ (Fig. 3).

The parthenium aqueous extract revealed four peaks. A broad peak in the 3371–3212 cm^−1^ ranges and sharp peaks at 1630, 1368, and 1209 cm^−1^. The FTIR spectrum of CuO NPs was observed at 3284, 1600, 1368, 1278, 1084, 820, 522, and 590 cm^−1^. The peak in the 3371–3212 cm^−1^ range indicates the presence of the O–H group typical of phenols in the extract was present in both the extract and CuO NPs spectra. These peaks correspond to previous study findings^63,64^. A peak at 1630 cm^−1^ and 1600 cm^−1^ for extract and nanoparticles are associated with a C=O bond stretching vibration. The C=O bond stretching vibration is typical of amides^65^. The peaks at 1368 cm^−1^ for the extract and CuO NPs spectra represent the amines' C–N stretching vibration^34^. A peak at 1084 cm^−1^ is associated with C–O stretching vibrations typical to flavonoids^34,63^. The characteristic peak at 522 cm^−1^ and 590 cm^−1^ indicated the formation of Cu–O stretching vibrations, confirming the formation of CuO NPs^34,35^. Therefore, from the FTIR analysis, it can be indicated that the phytochemicals present in the aqueous extract, such as phenols and flavonoids, were responsible for reducing and stabilizing metal ions to form CuO NPs.

### Scanning electron microscope analysis

**Figure 4:**
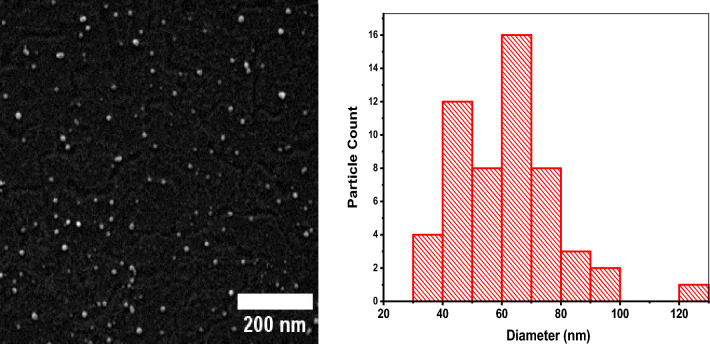
SEM micrograph and size distribution of CuO NPs.

The morphology and surface structure of the synthesized CuO NPs were analyzed using SEM, and obtained micrograph is shown in Fig. 4.

The shape of the nanoparticles was nearly spherical, with less evidence of agglomeration. Previous studies confirmed CuO NPs' spherical shape^66,67^. The average particle size determination using ImageJ software showed that the average particle size was 59.99 nm. The particle size distribution for CuO NPs obtained in this study agreed with previous studies^67,68^.

### Transmission electron microscope

**Figure 5:**
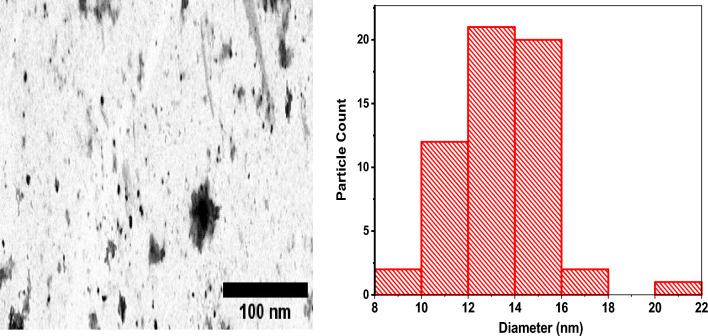
TEM micrograph and size distribution of CuO NPs.

The size of the synthesized copper oxide nanoparticles was also visualized using TEM micrographs, as depicted in Fig. 5.

The TEM micrographs revealed that the nanoparticles were nearly spherical^69^. The particle size distribution from the TEM micrograph was 8–22 nm which falls within the size range of CuO NPs obtained in previous studies^69,70^.

### X-ray diffraction analysis

**Figure 6:**
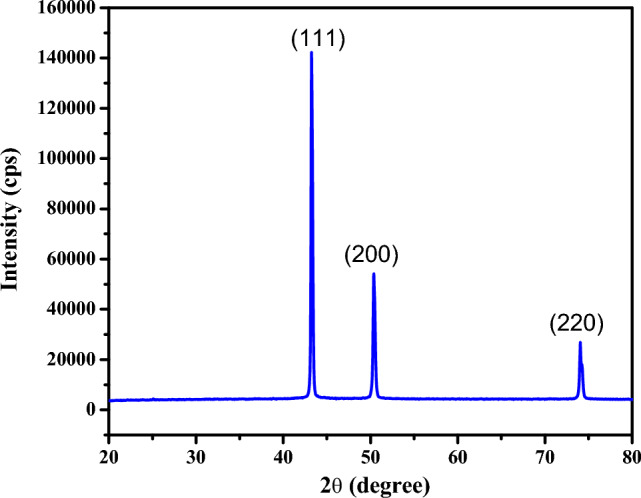
XRD pattern of CuO NPs.

The XRD diffraction pattern of CuO NPs is presented in Fig. 6.

X-ray diffraction analysis shows the presence of three prominent peaks at 2θ values in the range of 20° to 80° at 43.6°, 50.8°, and 73.5°. These 2θ values present (111), (200), and (220) crystallographic planes from the JCPDS database, respectively^38,41^. The XRD pattern confirmed the high crystallinity nature of CuO NPs^31^. The peaks can be indexed to be typical monoclinic structures of CuO NPs^34^. The average crystallite size was calculated using the Scherrer equation^34,43^ and was found to be 31.58 nm. Other researchers have confirmed the green synthesis of CuO NPs in this size range. 22.4 nm of CuO NPs were synthesized using *Bacopa monnieri* leaf extract^71^. The average size of CuO NPs was calculated as 29.7 nm in a green synthesis using *Curcuma longa* root extracts^31^. The d-spacing and lattice constant parameters were determined using Bragg’s law to be 0.2 nm and 0.34 nm, respectively. These results correspond to previous study^72^.

### Dynamic light scattering analysis

**Figure 7:**
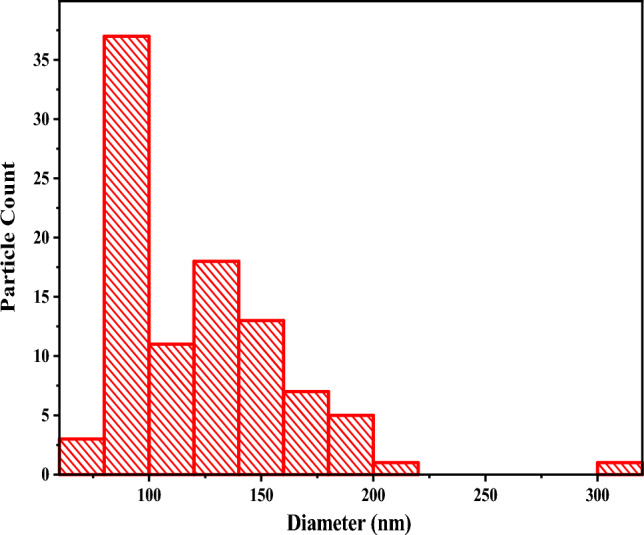
Size distribution of CuO NPs from DLS.

Dynamic light scattering was used to determine the size distribution of the synthesized copper oxide nanoparticles, and the results are depicted in Fig. 7.

The DLS histogram shows that the particles were larger and polydisperse compared to those observed in SEM micrographs which range from 20 to 120 nm (Fig. 4). The diameter of the particles was in the range of 0 to 200 nm, with a large population of nanoparticles having an average size below 100 nm. The polydispersity index (PDI) was 0.3 or less, which indicated that the individual size distribution was monodispersed^73^. As a result, individual monodispersity can be attributed to agglomeration or aggregation during the nanoparticle synthesis process^74^.

### Degradation studies of rifampicin using CuO NPs

#### Effect of CuO NPs dose on degradation efficiency

**Figure 8:**
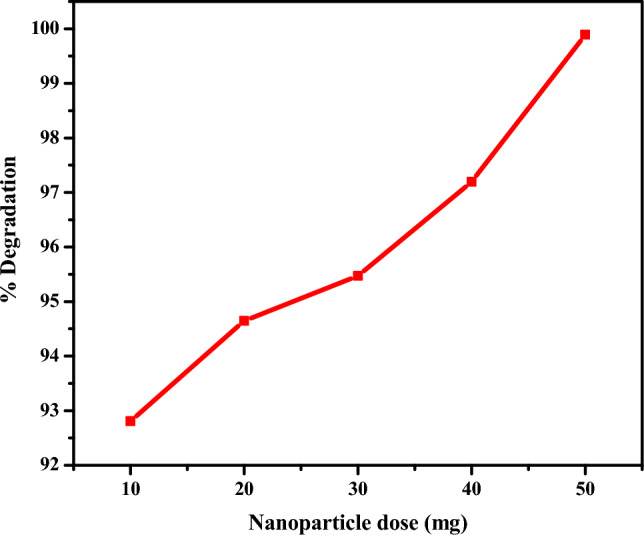
Effect of CuO NPs dose on degradation efficiency.

Figure 8 depicts the effect of CuO NPs dose on the degradation of the rifampicin antibiotic.

As can be seen in Fig. 8 as the dose of CuO NPs was increased from 10 to 50 mg, the percentage of degradation of rifampicin increased from 92.81 to 99.89%, respectively, within 210 min. The increase in the degradation percentage upon increasing the dose of CuO NPs can be attributed to the increased total surface area of the nanoparticles, which increases the active sites that interact with the rifampicin antibiotic^75,76^. Therefore, as the number of active sites on the surface of CuO NPs increases, more reactive radicals are generated, which are involved in the degradation of rifampicin, and the degradation efficiency improves^76,77^. It was observed that the intensity of absorption of rifampicin decreased as the amount of CuO NPs was increased. This supports the fact that more molecules of rifampicin were bound to the active sites of the CuO NPs, and their structure was destroyed quickly and faster as more active sites were available at higher doses of CuO NPs. The decrease in absorbance intensity at higher nanoparticle doses indicates the formation of intermediate degradation byproducts.

#### Effect of rifampicin concentration on degradation

**Figure 9:**
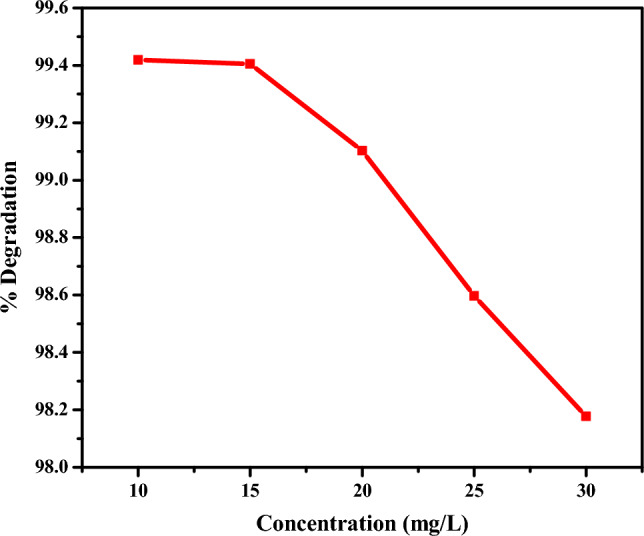
Effect of rifampicin concentration on degradation efficiency.

Figure 9 depicts the effect of the concentration of rifampicin antibiotic on the degradation efficiency using CuO NPs.

As the concentration of rifampicin increased from 10 to 30 mg/L, the percent of degradation of rifampicin decreased from 99.42 to 97.78%, respectively, within 12 min of the reaction. At higher rifampicin concentrations, all active sites of CuO NPs were occupied and saturated to react and degrade the rifampicin, resulting in less degradation percent at high concentrations of rifampicin^76,77^. All the active sites of CuO NPs were used at higher concentrations of rifampicin, and more molecules of rifampicin remained in the solution. Simultaneously, the generation of reactive species such as OH radical from the hydrogen peroxide in the solution becomes suppressed when the concentration of rifampicin solution is high^76^. Another possible reason for a decreasing degradation efficiency as the concentration of rifampicin increases results from the production of degradation byproducts which competes with the rifampicin molecules for the limited number of active sites on the surface of CuO NPs and the reactive radicals present. Regarding absorbance from the UV–Vis spectrum, it was observed that at higher concentrations of rifampicin, the absorbance intensity of rifampicin was higher, indicating minimal degradative interaction between rifampicin and CuO NPs^78,79^.

#### Effect of pH on degradation

**Figure 10:**
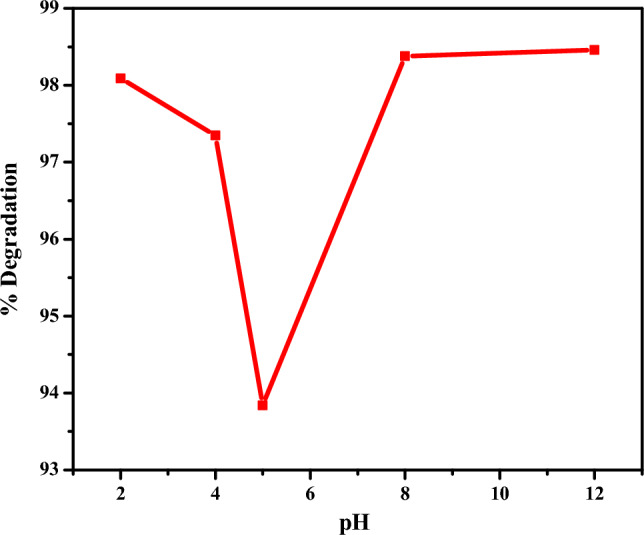
Effect of pH of rifampicin solution on degradation.

Figure 10 depicts the effect of varying pH on the efficiency of degradation of rifampicin using CuO NPs.

The pH of the rifampicin solution was varied using 0.1 M HCl and 0.1 M NaOH solutions. By varying the pH of the solution, the concentration of H^+^ and OH^−^ ions become affected, which are responsible for generating reactive species involved in the degradation process of rifampicin antibiotic. In acidic conditions at pH 2 and 4, the percentage degradation of rifampicin by CuO NPs was calculated and found to be 98.09% and 97.35%, respectively. In basic conditions, the degradation studies were carried out at pH 8 and 12, which yielded a degradation efficiency of 98.38% and 98.46%, respectively. Compared to the solution of rifampicin which was at pH 5 and had a degradation efficiency of 93.84%, it was observed that the rate of degradation of rifampicin increased significantly at both acidic and basic media. The increase in the efficiency of degradation in acidic media can be attributed to the generation of hydrogen radicals upon the interaction of hydrogen peroxide with CuO NPs^80^. Simultaneously, in acidic media, the nanoparticles become positively charged, which increases the rate of interaction of H_2_O_2_ with CuO NPs resulting in the generation of more H^+^ ions, and increasing the degradation of rifampicin^11,75^. The increase in efficiency of degradation of rifampicin in basic media can be attributed to the increase in OH^−^ ions leading to the generation of more hydroxyl radicals which activates the active sites of CuO NPs. The increase in hydroxyl radicals advances the interaction rate of nanoparticles with rifampicin solution leading to improved degradation efficiency compared to rifampicin solution at pH 5. The degradation of rifampicin can be described to be pH-dependent, and its alteration in the surface charge of the CuO NPs results in varying percentage degradation^81^.

#### Effect of time on degradation

**Figure 11:**
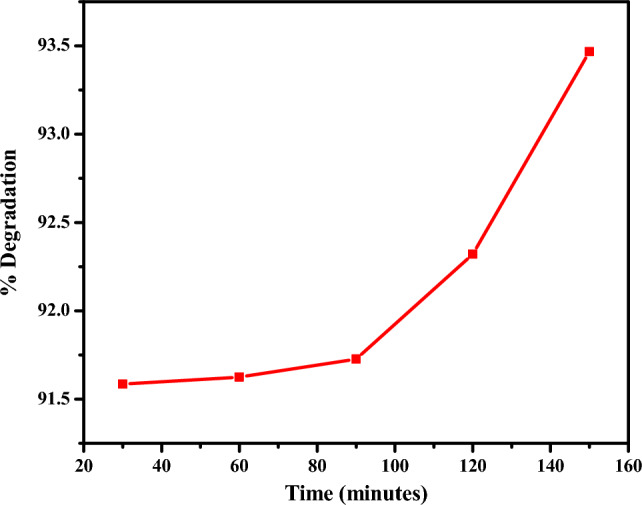
Effect of time on the rate of degradation of rifampicin.

Figure 11 depicts the effect on the interaction time of the rifampicin antibiotic with the CuO NPs.

The degradation of rifampicin was observed by measuring the intensity of absorption at regular time intervals. The decrease in absorption intensity at λ_max_ = 480 nm was correlated to the reduction in rifampicin concentration, which indicated the increasing degradation rate of rifampicin by CuO NPs with interaction time^82^. These results were consistent with previous studies which reported improved removal efficiency of levofloxacin antibiotic using CuO NPs from 27% in 15 min to 71% within 120 min^83^.

#### Effect of temperature on degradation

**Figure 12:**
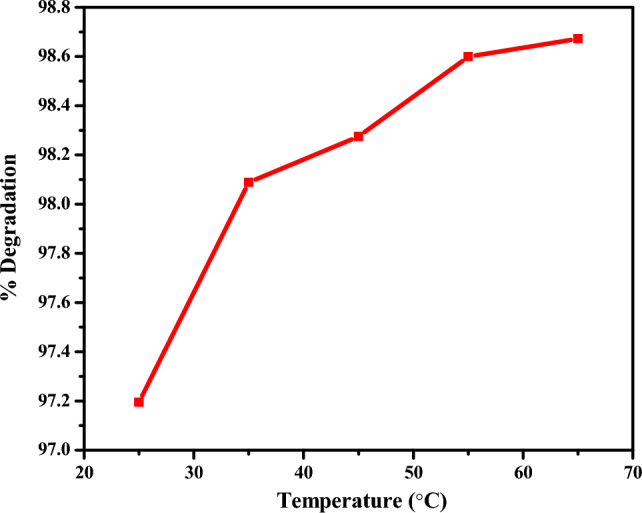
Effect of temperature on degradation efficiency.

Figure 12 depicts the observable changes in the effect of temperature on the percent of degradation of rifampicin using CuO NPs.

As the temperature increased from 25 to 65 °C, the percent of degradation of rifampicin increased from 97.19 to 98.76%, respectively. As reported in previous studies, increases in temperature improve the kinetics of degradation because of an increase in Brownian motion^75,84^. It was also observed through the change in absorbance of rifampicin, which was reduced upon increasing the temperature after 24 min^11^.

**Figure 13:**
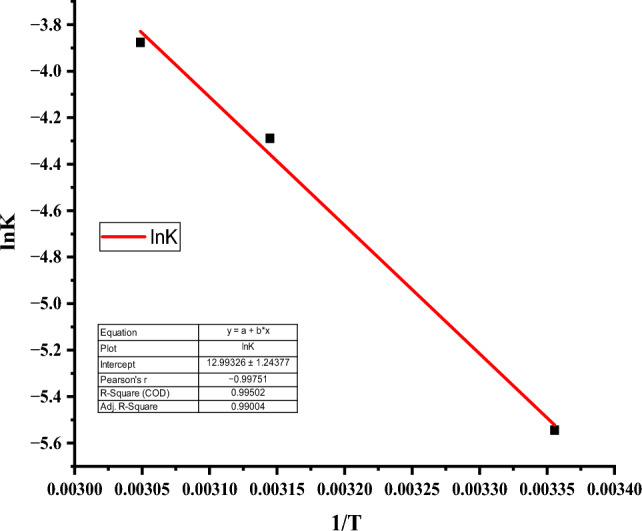
Van’t Hoff plot for degradation of rifampicin.

**Figure 14:**
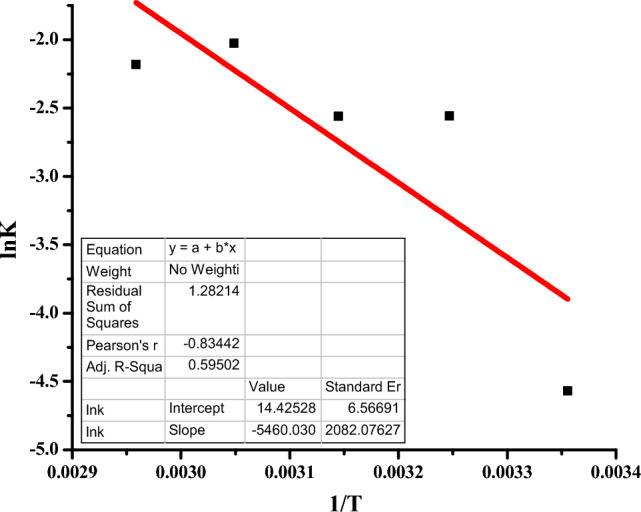
Arrhenius plot of lnk against 1/T to obtain activation energy of the degradation reaction of rifampicin.

The change in heat and entropy of the degradation reaction of rifampicin using CuO NPs was determined from the slope and intercept values, respectively, of Van't Hoff's plot of $ln {k}_{eq}$ against $\frac{1}{T}$, and the results are depicted in Fig. 13. The activation energy of the degradation process was also determined using Arrhenius plot of lnk against 1/T and the results are represented in Fig. 14.

Van't Hoff's equation relate the change in equilibrium constant with a decrease in the concentration of rifampicin with increasing temperature. It was observed that at higher temperatures, the rifampicin degradation rate was higher (Fig. 12), indicating that the reaction was endothermic^81^. Temperature plays a significant role in enhancing the degradation rate of the antibiotic. The entropy of an endothermic reaction decreases when the reaction temperature increases due to the formation of degradation byproducts. For this reaction, the change in the heat was calculated from the experimental data to be 45.87 kJ mol^−1^, while the change in entropy of the reaction was 108.03 J K^−1^^11^. The value of activation energy (E_a_) of rifampicin degradation was calculated from the slope of the Arrhenius plot of lnK against 1/T by following pseudo-second-order kinetics established to occur during the degradation of rifampicin antibiotic by CuO NPs. The value of E_a_ was determined to be 454 kJ mol^−1^.

### Kinetics of degradation of rifampicin

**Figure 15:**
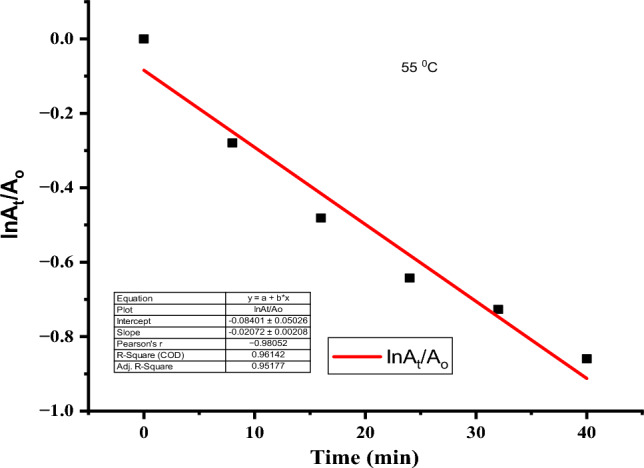
Pseudo-first order plot at 55 °C.

**Figure 16:**
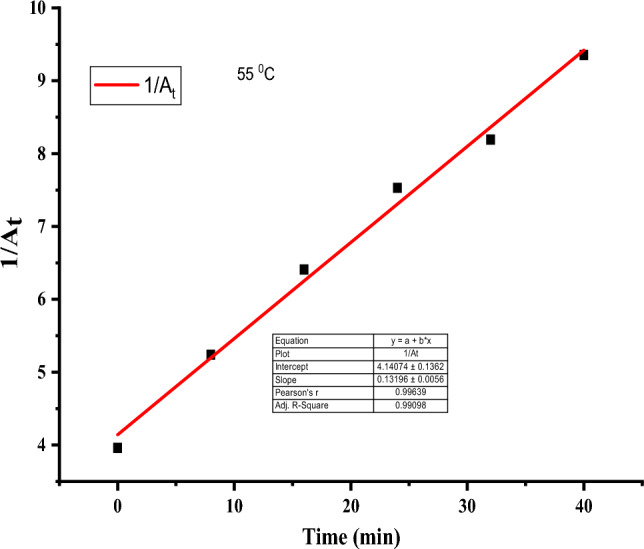
Pseudo-second order plot at 55 °C.

To understand the degradation kinetics of rifampicin, the kinetic models were fitted using pseudo-first order and pseudo-second order at 298, 308, 328, 328, and 338 K temperatures, as depicted in Table 2, Figs. 15, and 16.

**Table 2:** Kinetic models fitting of experimental data at different temperatures.

Temperature (K)	Pseudo-first order		Pseudo-second order	
	Rate constant (min^−1^)	R^2^	Rate constant (min^−1^)	R^2^
298	− 0.00391	0.99382	0.01038	0.9917
308	− 0.01424	0.99516	0.07749	0.98263
318	− 0.01372	0.98761	0.07725	0.99477
328	− 0.02072	0.96142	0.13196	0.99278
338	− 0.01736	0.9655	0.11281	0.99406

Under varying temperature conditions, the kinetic data fitted pseudo-second order because the R^2^ (correlation coefficient) values were ≥ 0.98 for all temperature conditions, which were higher than pseudo-first-order R^2^ values. The increase in the rate constants indicated that the reaction was endothermic, consistent with previous findings on the degradation of rifampicin antibiotic^11^.

### Degradation efficiency of rifampicin at optimal condition

**Figure 17:**
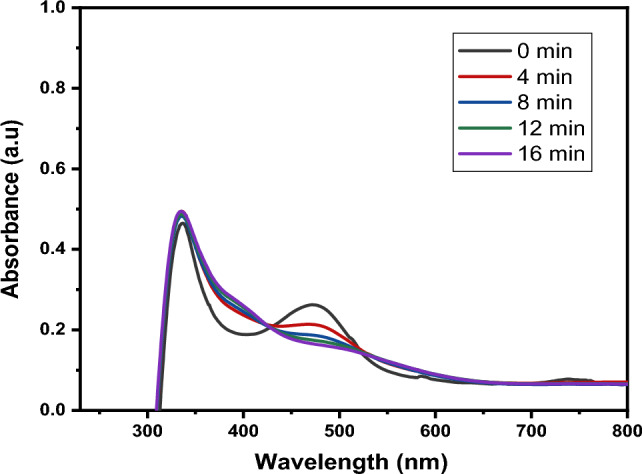
Degradation of rifampicin at optimal conditions at pH 8.

**Figure 18:**
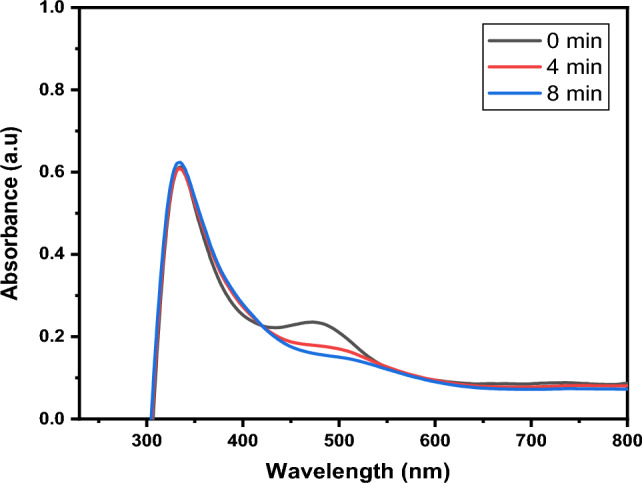
Degradation of rifampicin at optimal conditions at pH 2.

Figures 17 and 18 depict the degradation efficiency at optimum conditions (10 mg/L rifampicin concentration, 65 °C, and 50 mg CuO NPs, pH 2 and 8).

The percentage degradation of rifampicin at pH 8 using the mentioned optimal conditions was calculated and found to be 98.37%, achieved at 16 min. Using the same optimal parameters at acidic pH 2, the degradation efficiency was calculated to be 98.43%, achieved after 8 min. It can be concluded that at the highest dose of CuO NPs (50 mg), more active sites are available to react with the rifampicin molecules, while acidic (pH 2) and basic (pH 8) and increased temperature conditions increase the generation of hydrogen and hydroxyl radicals which further activates the surface of the nanoparticles leading to enhanced degradation efficiency. These optimal conditions increase the generation of radicals, improving the rate at which the rifampicin antibiotic is degraded^11^.

### Mechanism of degradation of rifampicin using CuO NPs

**Figure 19:**
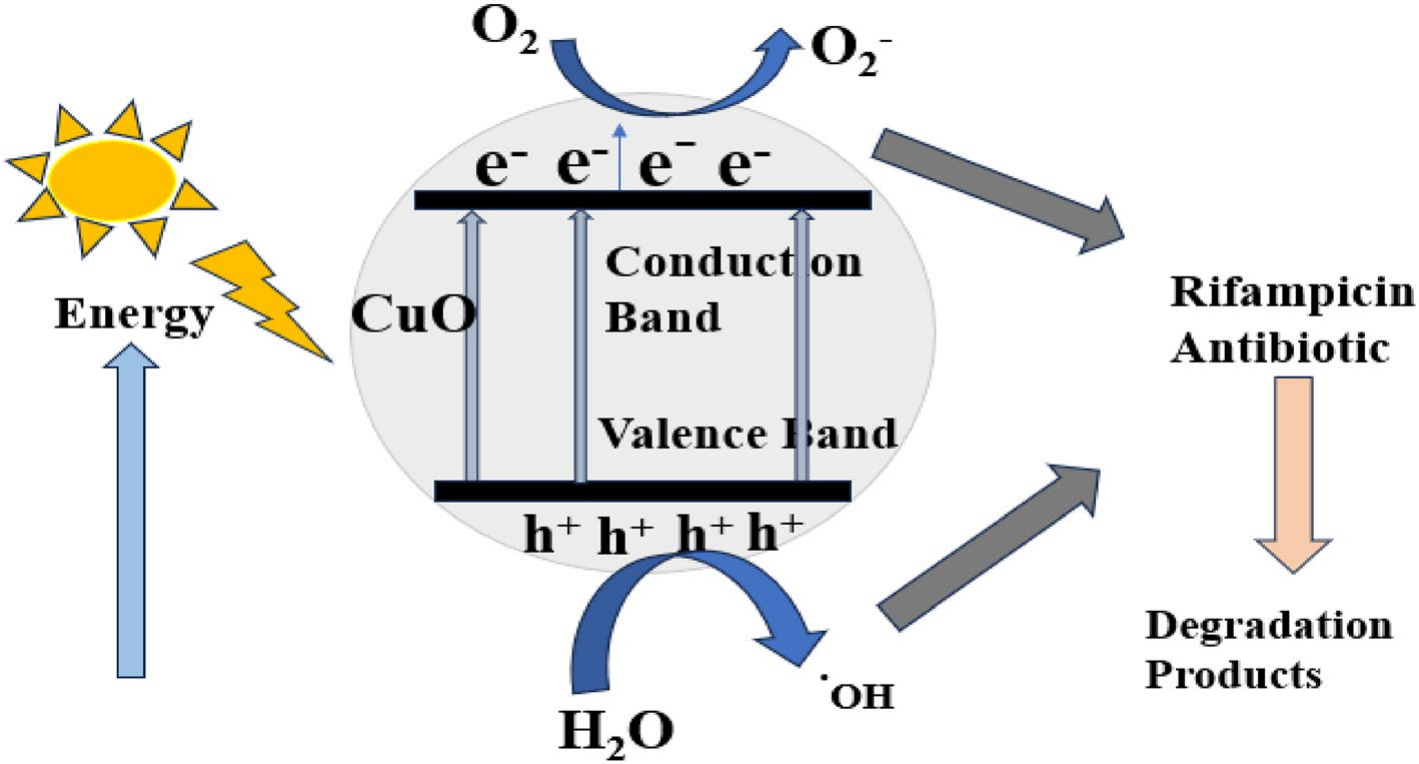
Probable degradation mechanism of rifampicin antibiotic using CuO NPs.

Several studies have reported the mechanism of degradation of antibiotics using metallic nanoparticles^41,77–79^. The presence of incident light on the surface of CuO NPs results in a hole in the valence band (VB). This creates a positive charge on the VB $\left({\mathrm{h}}_{\mathrm{VB}}^{+}\right)$. As a result, the electron is excited from VB to conduction band CB $({\mathrm{e}}_{\mathrm{CB}}^{-})$^85^. The presence of excited electrons on the CB reduces oxygen molecules to oxygen radicals, while the water molecules in the reaction are oxidized to hydroxyl radicals^86,87^. Figure 19 illustrates the mechanism of degradation of rifampicin using CuO NPs. The mechanisms of degradation of MB dye by ZnO NPs can also be described using the steps shown in the equations below.

2 $$\mathrm{CuO \; NPs}+\mathrm{ hv }\to \mathrm{CuO \; NPs }\left({\mathrm{e}}_{\mathrm{CB}}^{-}\right)+\mathrm{CuO \; NPs }\left({\mathrm{h}}_{\mathrm{VB}}^{+}\right),$$

3 $$\mathrm{CuO \; NPs }\left({\mathrm{e}}_{\mathrm{CB}}^{-}\right)+{\mathrm{O}}_{2}\to \mathrm{CuO \; NPs }+ {\mathrm{O}}_{2}^{\cdot -},$$

4 $${\mathrm{O}}_{2}^{\cdot -} +{\mathrm{H}}^{+ }\to {\mathrm{HO}}_{2}^{.},$$

5 $${\mathrm{HO}}_{2}^{.} + {\mathrm{H}}^{+} + {\mathrm{O}}_{2}^{\cdot -}\mathrm{ or \; CuO NPs }\left({\mathrm{e}}_{\mathrm{CB}}^{-}\right) \to {\mathrm{H}}_{2 }{\mathrm{O}}_{2} + {\mathrm{O}}_{2}+\mathrm{CuO \; NPs},$$

6 $$\mathrm{CuO \; NPs }{(\mathrm{h}}_{\mathrm{VB}}^{+}) + {\mathrm{H}}_{2}\mathrm{O }\to \mathrm{CuO \; NPs }+ {\mathrm{H}}^{+ }+ {\mathrm{HO}}^{\cdot },$$

7 $$\mathrm{CuO \; NPs }{(\mathrm{h}}_{\mathrm{VB}}^{+}) + {\mathrm{OH}}^{-} \to \mathrm{CuO \; NPs }+ {\mathrm{OH}}^{\cdot },$$

8 $$\mathrm{Rifampicin \; antibiotic }+{\mathrm{OH}}^{\cdot } \; \mathrm{ or } \; {\mathrm{O}}_{2}^{\cdot -}\to \mathrm{degradation \; products}.$$

### FTIR analysis of CuO NPs after degradation studies

**Figure 20:**
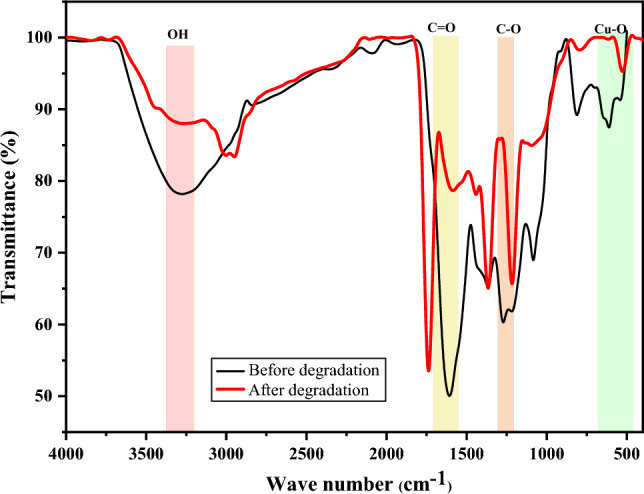
FTIR spectra of CuO NPs before and after degradation of rifampicin.

Figure 20 depicts FTIR spectra showing the changes in CuO NPs after degradative interaction with rifampicin.

As shown in Fig. 20, slight changes were observed in the FTIR spectra of CuO NPs after its use in the degradation of rifampicin antibiotic. Shifts in the wavenumber of the functional groups were observed after the CuO NPs interacted with rifampicin. The broadband typical of an OH group shifted from 3270 cm^−1^ to two peaks at 3288 cm^−1^ and 2949 cm^−1^ after degradation were slightly narrow than before degradation studies. The band typical of C=O groups shifted from 1609 cm^−1^ before degradation to 1737 cm^−1^ after degradation. The shift of the C–O was observed from 1271 cm^−1^ before degradation to 1209 cm^−1^ after degradation studies. The transitions were attributed to the adsorption of rifampicin on the surface of CuO NPs, which altered the frequencies. The peaks assigned to Cu–O functional group exhibited a slight shift from two peaks at 590 cm^−1^ and 522 cm^−1^ to a single peak after degradation at 528 cm^−1^. These changes in the peaks of CuO NPs before and after degradation studies of rifampicin demonstrate that the functionality of the CuO NPs was not lost by their degradative ability in removing antibiotics^84^.

### Recyclability potential of CuO NPs in the degradation process

**Figure 21:**
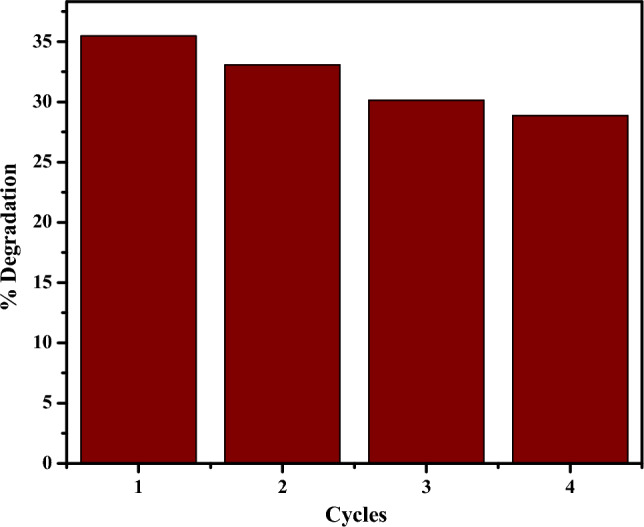
Recycle and reuse of CuO NPs in degradation of rifampicin in four cycles.

The recycle and reuse ability of CuO NPs in the degradation of rifampicin antibiotic was investigated in four cycles for 90 min each. After each use, the nanoparticles were decanted, washed with water, and dried in an oven at 60 °C for 6 h, as previously described^41,71^. The dry nanoparticles were then used in the subsequent degradation cycle with fresh 10 mg/L rifampicin solution. The degradation efficiency was calculated, and the results were plotted as shown in Fig. 21.

The degradation efficiency of CuO NPs on rifampicin antibiotic was observed to have declined from 35.48% (first cycle) to 28.87% (fourth cycle). The decrease in the degradation efficiency in each subsequent recycling cycle can be attributed to the adsorption of rifampicin antibiotics on the active sites of the CuO NPs, which deactivates the catalytic property of the nanoparticles^41,71^. In addition, the decrease in degradation efficiency of CuO NPs as the number of cycles increases can be supported by the fact that CuO NPs lose their catalytic performance during separation and drying steps^72,88^. This study's recyclability findings are consistent with previously reported studies^72,88^.

### Comparison with conventional antibiotic degradation methods

The effectiveness of CuO NPs in the degradation of rifampicin antibiotic was compared with other catalysts used in the degradation of antibiotics and summarized in Table 1. In this study, CuO NPs proved to have improved degradation efficiency in removing rifampicin, making the nanoparticles promising for application in the degradation of other antibiotics.

## Conclusion

The present study has described a cost-effective and environmentally friendly green synthesis of CuO NPs using *Parthenium hysterophorus* plant aqueous extracts as reducing and capping agents. The CuO NPs were characterized using UV–Vis spectroscopy, FTIR, SEM, TEM, XRD, and DLS analytical techniques. The UV–Vis spectrum revealed an SPR band at 340 nm attributable to CuO NPs. The FTIR analysis showed the presence of secondary metabolites in the extracts, which were responsible for reducing copper ion solutions into CuO NPs with characteristic stretching vibration at 522 cm^−1^. SEM and TEM micrographs confirmed the spherical shape of CuO NPs. The XRD analysis confirmed the crystallinity of CuO NPs, and crystallite size was calculated to be 31.58 nm. DLS studies revealed polydispersity of CuO NPs. CuO NPs showed to be effective in degrading rifampicin antibiotics, with a degradation efficiency of over 98% being reported at optimal conditions. The rifampicin antibiotic degradation follows a pseudo-second-order kinetic model. Therefore, an eco-friendly and cost-effective *Parthenium hysterophorus*-mediated green synthesis of CuO NPs can be promising in the degradation of antibiotics in wastewater treatment plants and serve to address the increasing water pollution challenges. Future research should focus on the usability of CuO NPs in large-scale wastewater treatment and identifying actual degradation products and their implications for the ecosystem. We also note that toxic degradation products on the environmental compartments can further exacerbate water pollution hence the need to prevent their occurrence through encapsulation or making magnetic nanocomposite. This way, the safety of nanoparticles can be enhanced as they address environmental pollution.

## Data Availability

The data associated with this research study that is enough to draw the results and conclusions have been provided within the manuscript. However, all datasets have been deposited in the public repository, Zenodo, and are accessible via the link https://doi.org/10.5281/zenodo.8116191.
